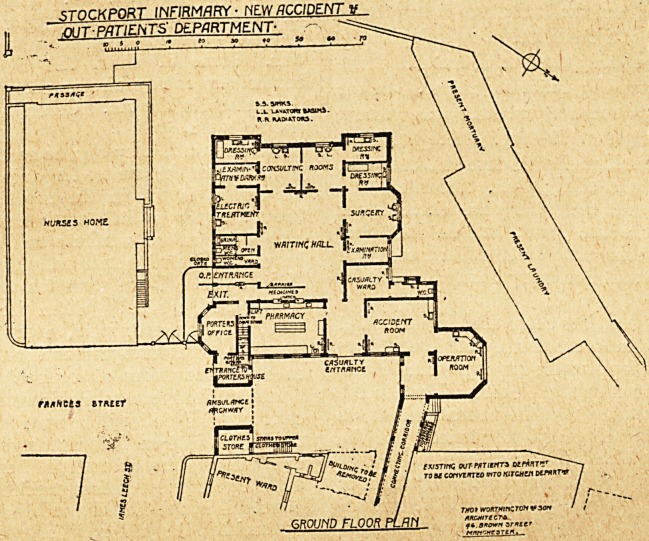# Stockport Infirmary

**Published:** 1919-06-07

**Authors:** 


### HOSPITAL ARCHITECTURE AND CONSTRUCTION.

### STOCKPORT INFIRMARY.

### New Accident and Out-Patient Department.

This recent addition to the Stockport Infirmary has been erected on a vacant site between the Nurses' Home and the laundry to the south-west of the main hospital, with which it is connected by a corridor.

The entrance for ambulances and for casualties faces the end of Frances Street, while the entrance for out-patients faces James Leech Street, which meets Frances Street at a right-angle. The accident department includes a large receiving-room, an operation-room, and a small ward with w.c. and sink attached.

There is only one door to the out-patient department, which serves for entrance as well as for exit; this opens on to an enclosed open space which is overlooked by the windows in the porter's lodge. Beyond the latter two doors are found, the first being for exit, the second for entrance, a partition separating the two. On the plan there is nothing to indicate any means of preventing the incoming patients using the exit passage, but it is presumed that some kind of automatic barrier is provided for the purpose.

There is the usual large central waiting-hall, off which are arranged two consulting - rooms, each with its dressing-room attached, a room for electric treatment, with a dark room, a surgery with dressing- and examination-rooms, a pharmacy, and separate sanitary offices for both sexes.

The dressing- and examination - rooms are planned so that two of them can be used in connection with either the electric department or one of the consulting-rooms, or with the surgery or the other consulting-rooms.

The department seems rather limited in size for so large a town as Stockport, and the absence of any provision for isolation needs explanation.

The architects are Messrs. Worthington and Son, of Manchester.

## Figures and Tables

**Figure f1:**